# Neural Radiance Fields-Based 3D Reconstruction of Power Transmission Lines Using Progressive Motion Sequence Images

**DOI:** 10.3390/s23239537

**Published:** 2023-11-30

**Authors:** Yujie Zeng, Jin Lei, Tianming Feng, Xinyan Qin, Bo Li, Yanqi Wang, Dexin Wang, Jie Song

**Affiliations:** College of Mechanical and Electrical Engineering, Shihezi University, Shihezi 832003, China; 20212009014@stu.shzu.edu.cn (Y.Z.); jinlei@shzu.edu.cn (J.L.); ftm@stu.shzu.edu.cn (T.F.); libo@stu.shzu.edu.cn (B.L.); 20212009001@stu.shzu.edu.cn (Y.W.); 20212009006@stu.shzu.edu.cn (D.W.); 20212009017@stu.shzu.edu.cn (J.S.)

**Keywords:** FPLIR, PTLs, 3D reconstruction, NeRF, progressive motion sequences

## Abstract

To address the fuzzy reconstruction effect on distant objects in unbounded scenes and the difficulty in feature matching caused by the thin structure of power lines in images, this paper proposes a novel image-based method for the reconstruction of power transmission lines (PTLs). The dataset used in this paper comprises PTL progressive motion sequence datasets, constructed by a visual acquisition system carried by a developed Flying–walking Power Line Inspection Robot (FPLIR). This system captures close-distance and continuous images of power lines. The study introduces PL-NeRF, that is, an enhanced method based on the Neural Radiance Fields (NeRF) method for reconstructing PTLs. The highlights of PL-NeRF include (1) compressing the unbounded scene of PTLs by exploiting the spatial compression of normal $L^{\infty }$; (2) encoding the direction and position of the sample points through Integrated Position Encoding (IPE) and Hash Encoding (HE), respectively. Compared to existing methods, the proposed method demonstrates good performance in 3D reconstruction, with fidelity indicators of PSNR = 29, SSIM = 0.871, and LPIPS = 0.087. Experimental results highlight that the combination of PL-NeRF with progressive motion sequence images ensures the integrity and continuity of PTLs, improving the efficiency and accuracy of image-based reconstructions. In the future, this method could be widely applied for efficient and accurate 3D reconstruction and inspection of PTLs, providing a strong foundation for automated monitoring of transmission corridors and digital power engineering.

## 1. Introduction

PTLs play a crucial role in meeting daily electricity demands for various aspects of life and work. However, their distribution across diverse terrains such as mountains, plains, deserts, or other natural environments makes them susceptible to environmental impacts that can lead to problems like power line breakage, damage, and erosion. These issues have the potential to cause large-scale blackouts and result in significant national economic losses. Therefore, conducting regular inspections becomes indispensable to guarantee the safety of PTLs [1,2,3].

There are four types of inspections: manual inspections, vehicle inspections, airborne inspections, and robot inspections. Among them, manual inspections for PTLs are always costly, dangerous, and prone to false results [4,5,6]. Vehicle inspections and airborne inspections use vehicles or aircraft as carrying platforms, but are limited to inspecting PTLs in urban areas [7]. Although manual inspections currently dominate the field, in contrast, robot inspections present enhanced convenience, safety, and flexibility, positioning them as the future trend [8]. In current robot inspections, a critical task is the 3D reconstruction of PTLs [9,10]. The 3D reconstruction of PTLs enables efficient line layout planning, monitoring, and management of the power system with its surroundings, enhancing safety and reliability in the power system while optimizing the line layout and reducing resource wastage and environmental pollution. It can also reduce the need for on-site inspections, especially in difficult-to-reach areas. This not only improves efficiency, but also reduces maintenance costs and personnel safety risks. Therefore, it is of significant value in the field of power engineering. In addition, through 3D reconstruction, the fault points can be quickly located and effectively repaired in emergency situations, improving operational efficiency, maintenance management, and safety risk control [11]. In the future, 3D reconstruction of PTLs will become an essential technology in supporting the development of power engineering [12].

Currently, there are two main categories of 3D reconstruction techniques for PTLs: LiDAR-based and image-based methods. LiDAR-based methods offer high data acquisition efficiency, modeling quality and measurement accuracy, and are less affected by various lighting conditions. However, they are expensive and not suitable for large-scale applications [13]. On the other hand, image-based methods are less expensive, offer good flexibility, and provide impressive 3D visualization. Nevertheless, these methods tend to have lower accuracy and require more complex algorithms to overcome their limitations [14,15,16]. Conventional image-based methods for 3D reconstruction have certain limitations regarding accuracy and complexity. They rely on capturing images from a single angle during inspection, which decreases the overall reconstruction quality [17,18]. In addition, challenges like unknown depth information in unbounded scenes, thin structures of power lines, and feature-point matching further complicate the reconstruction task for PTLs [19,20].

To address the mentioned challenges, this paper proposes a method for acquiring motion sequence images and establishing PTL reconstruction datasets. Additionally, this approach improves the spatial compression and coding techniques of the original NeRF to reconstruct PTLs. Overall, it eliminates the requirement of depth information and enables a more efficient reconstruction method for PTLs based on images and camera poses, supported by supervised scene learning.

**Figure 1:**
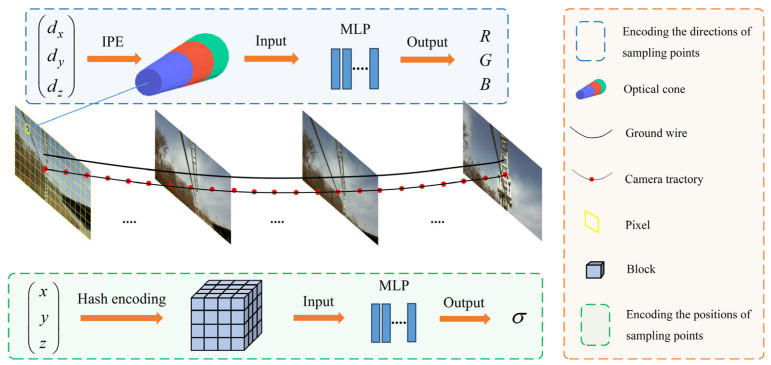
PL-NeRF position-encoding system.

The main contributions of this study are as follows:

(1) Using progressive motion sequences, datasets are established for the 3D reconstruction of PTLs. To achieve accurate position estimation over long trajectories, the trajectories are segmented to dynamically generate the neural radiance field. This serves as the basic dataset for the progressive motion sequence images, which consists of multiple views of PTLs captured by a vision acquisition system;

(2) Considering the long and narrow spatial structure of the PTLs, the spatial compression method in the original NeRF has been further improved to enhance scene reconstruction. Instead of using the NDC method, this paper employs the $L^{\infty }$ norm scene contraction method to shrink the unbounded scene into a fixed-size bounded space, aligning the scene model more effectively with the hash code, thereby improving the reconstruction efficiency of PTLs;

(3) Considering the thin structure of the power line, the position encoding (PE) method has been improved to better encode sample points in the original NeRF. IPE and HE are used for the direction and position of the sample points, respectively, as shown in Figure 1. IPE uses truncated cones to segment the sample points, and then generates the encoding results by calculating the integral and expected value on a circular table in Gaussian space. The method solves the problem of jagged and discontinuous results with PE; thus, it can effectively reconstruct continuous PTLs and improve the reconstruction quality. Meanwhile, HE enhances the efficiency of PTL reconstruction by adaptively focusing on the effective region.

The PL-NeRF position-encoding system is shown in Figure 1. We train two MLPs: an MLP for points’ colors and another one for points’ positions. The former has 4 layers with 256 hidden units per layer, while the latter has 8 layers with 256 hidden units per layer.

The rest of this paper is organized as follows. Section 2 provides an overview of the current research and related work. Section 3 describes the process of building the dataset for progressive motion sequence of PTLs. Section 4 presents the proposed method used to reconstruct PTLs. Section 5 conducts a comparison test to verify the effectiveness and feasibility of the proposed method. Section 6 discusses the experimental results and presents both limitations and prospects of the proposed method. Section 7 summarizes the results and engineering value of PL-NeRF.

## 2. Related Works

This paper introduces a PL-NeRF method for 3D reconstruction, focusing specifically on progressive motion sequence images. This section discusses three aspects within related works: (1) image-based 3D reconstruction of PTLs; (2) NeRF; (3) multi-view images.

### 2.1. Image-Based 3D Reconstruction of PTLs

The methods for reconstructing PTLs based on images can be categorized into two categories: traditional methods and deep learning methods. Their respective descriptions and characteristics are listed in Table 1.

Traditional methods generally meet the basic requirements but still have limitations, including difficulties in accurately capturing complex lighting conditions or handling intricate geometric details that require manual adjustments for each specific scenario. In contrast, deep learning methods offer greater flexibility and intelligence for generating high-quality and realistic 3D effects. Therefore, they may become a dominant research direction in the future. In line with this trend, this paper proposes an improved deep learning method called PL-NeRF, which is based on the original NeRF method [21].

**Table 1:** Image-based 3D reconstruction methods for PTLs.

Methods	Classification	Description	Advantages	Limitations	Literatures
Traditional	SfM	Reconstruct PTLs through feature matching and triangulation of the aerial images captured by UAVs.	High flexibility.	Limited accuracy, distorted model, limited ability in processing large-scale data, requiring a significant amount of image and computing resources.	[4,9,10,14,22,23]
	SLAM	Continuously update the map by processing a large amount of images data in real-time and calculate camera poses.	More emphasis on localization rather than 3D reconstruction.	Requiring for dynamic changes in the environment, also requiring the device have the ability to capture images in real-time.	[24,25,26]
Deep learning	/	Use neural networks to learn the 3D geometric information about PTLs from images without human intervention. By constructing a deep learning model, the input 2D images of the PTLs are converted into a 3D model.	Fast training speed, strong processing ability, and high accuracy.	Requiring a large amount of data and model training.	[23,27,28,29,30]
	Neural Radiance Fields, NeRF	A deep learning model is used to learn the radiance field representation of the scene and rendered using a ray-tracing algorithm.	Low cost and realism.	Slow rendering and poor reconstruction of complex scene details.	[21]

### 2.2. Neural Radiance Fields

The NeRF algorithm, developed in recent years, has significant research value and promising application prospects in image-based 3D reconstruction. It was first proposed by Mildenhall et al. in 2019 [21], who used a deep learning model to acquire a radiance field representation of the scene, followed by rendering using a ray-tracing algorithm. This technique has made a breakthrough and has attracted widespread attention in the field of 3D reconstruction. However, the original NeRF had slow reconstruction speed and limitations in reconstructing complex scene details. To address these issues, Mildenhall et al. [31] further introduced NeRF++ in 2020, which used multiple decoders to address different spatial scales, resulting in an improvement in the rendering speed. In the same year, Alex Yu et al. [32] proposed pixelNeRF to capture tiny features and textures in the scene, enabling detailed scene reconstruction. Recognizing the impact of illumination in these methods, NeRV [33] was proposed by Pratul P. Srinivasan et al. in 2021. It employs a random variational autoencoder to learn scenes, which can change lighting conditions and synthesize new perspectives in existing images, ultimately improving rendering quality. Over the past four years, many innovative techniques and application studies have emerged based on NeRF framework. For example, mip-NeRF [34] has improved its anti-aliasing capabilities, while PointNeRF [35] enables high-quality model acquisition without dense sampling. FastNeRF [36] is a fast and accurate neural rendering technology, while Instant-NGP [37] is a fast reconstruction technology that supports real-time inter-action and dynamic modification. Furthermore, NeuRay [38] is a ray-tracing technique that can account for occlusion issues and generate more realistic images. MvsNeRF [39] is another technique for reconstructing radiance fields from multi-view stereo images. There are also NeRF- [40] and SCNeRF [41], which can model radiance fields and perform pose estimation without camera parameters.

### 2.3. Multi-View Images

Single-view information is insufficient to accurately reconstruct a complete 3D scene due to many factors, such as the surface obstructions, the effect of lighting angle, and in-tensity on the images. In contrast, multi-view images can overcome these limitations to effectively process complex scenes and achieve a more comprehensive reconstruction. Therefore, multi-view images play a crucial role in high-quality image-based 3D reconstruction. At present, there are three types of multi-view data collection methods in the field of 3D reconstruction based on camera motion trajectory: free, forward facing, and object-centric. The methods, such as Structure-from-Motion (SFM) [42], Multi-view Stereo (MVS) [43], and F^2^-NeRF [44], are the representations of free camera trajectory. The LLFF method [45] represents forward-facing camera trajectory. The methods, such as NeRF [21], Mip-nerf 360 [46], Anything-3D [47], and OmniPhotos [48] represent object-centric ones. Their respective advantages and disadvantages are listed in Table 2.

The method of multi-view data collection should consider many factors, such as the complexity of the scene, the limitations of the equipment, and the requirements for 3D modeling accuracy. Considering that PTLs are detected along the power transmission lines in our study, which helps in reducing the cost and energy consumption by extending the detection distance, we have, ultimately, chosen the forward-facing collection method.

## 3. Progressive Motion Sequence Images

### 3.1. Vision Acquisition System

**Figure 2:**
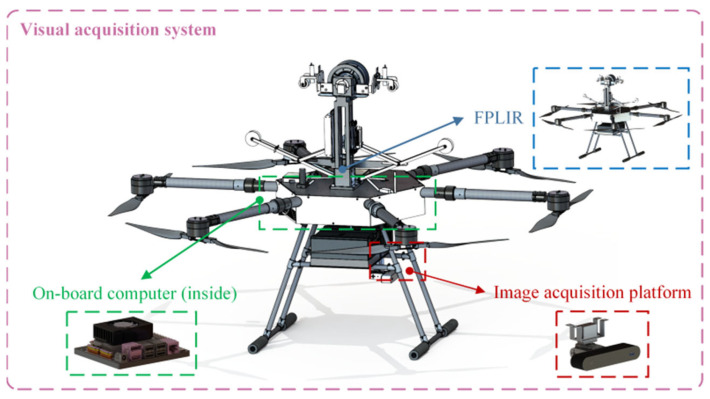
Visual acquisition system.

**Figure 3:**
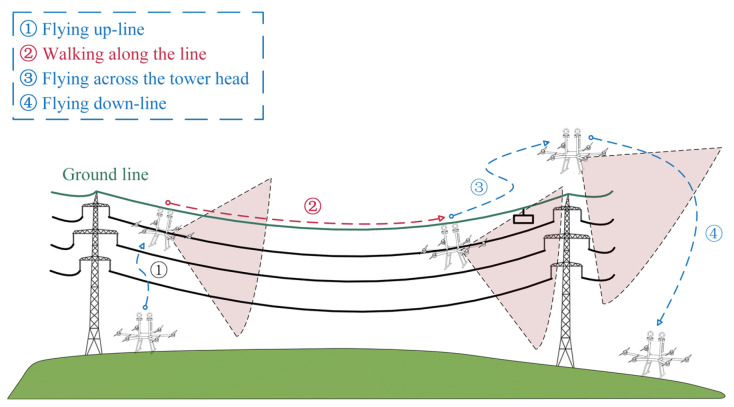
The process of PTL inspection using FPLIR.

The vision acquisition system mainly consists of an image acquisition platform, a developed FPLIR [3,49], and an on-board computer, as shown in Figure 2. The entire inspection process of the FPLIR is illustrated in Figure 3, which includes taking off from the ground, approaching the ground wire, landing on the ground wire, rolling along the ground wire, flying over obstacles and tower heads, and, finally, leaving the ground wire to land on the ground. Multi-view images are captured by the image acquisition platform during the phase of rolling along the ground wire.

**Figure 4:**
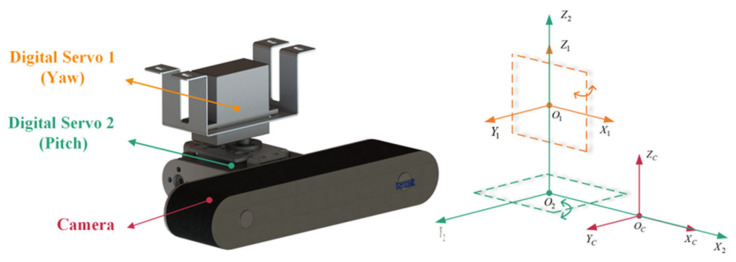
The structural diagram of image acquisition platform.

The multi-view motion sequence images of PTLs captured by the image acquisition platform on the FPLIR are at a close range, which is necessary for PTL reconstruction. The platform consists of a camera and two digital servos, as shown in Figure 4, with detailed parameters given in Table 3.

**Figure 5:**
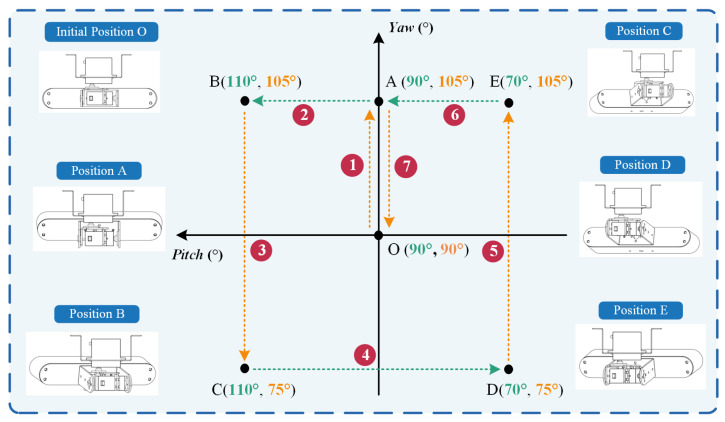
The motion demonstration process of the image acquisition platform.

**Figure 6:**
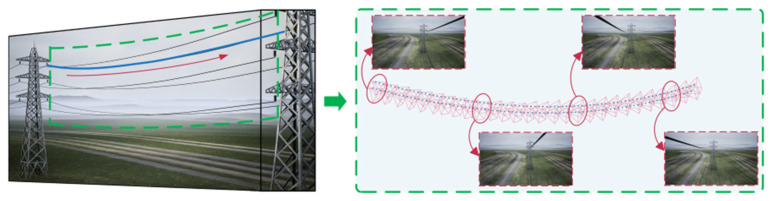
Camera trajectory.

Figure 5 illustrates the motion demonstration process of the platform, which exhibits periodic cyclical motion. Each cycle comprises seven stages (① to ⑦), with each stage taking the same amount of time, for a total of 49 s. In the initial state, Digital servo 1 and Digital servo 2 are both positioned at an angle of 90°, with movement ranges of 75~105° and 70°~110°, respectively. During each cycle, the two digital servos rotate sequentially (stages ①, ③, ⑤, and ⑦ represent the motion of Digital servo 1; stages ②, ④, and ⑥ represent the motion of Digital servo 2), alternating and continuing until the end of the walking phase. Figure 6 shows the relevant motion trajectories and processes from different perspectives. The arrow indicates the image captured at any frame during the process.

### 3.2. Dataset Construction

#### 3.2.1. Collection Sites and Path

**Figure 7:**
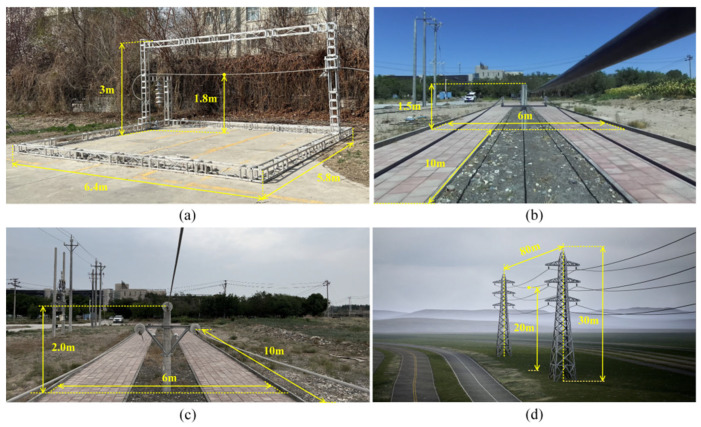
Test sites. (**a**) Site A; (**b**) Site B; (**c**) Site C; (**d**) Site D.

**Figure 8:**
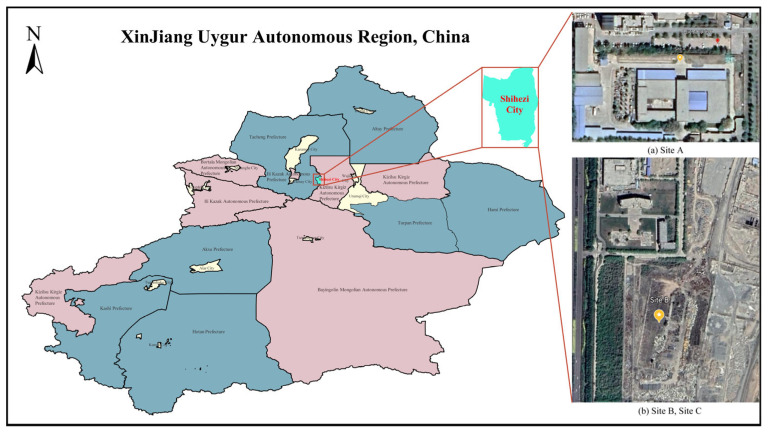
Locations of Site A, Site B, and Site C.

The four different test sites are shown in Figure 7. Site A is a constructed test site with dimensions of 5.8 m × 6.4 m, a tower height of 3.0 m, and a distance of 1.8 m between the lowest point on the power line and the ground. Site B is a PTL inspection training site with dimensions of 6 m × 10 m, a tower height of 1.5 m, and a distance of 1.2 m between the lowest point on the ground wire and the ground. Site C is another PTL inspection training site, covering an area of 6 m × 10 m, with a tower height of 2.0 m and a distance of 1.2 m between the lowest point on the ground wire and the ground. Finally, Site D is a virtual constructed scene model, covering an area of 12 m × 80 m, with a tower height of 30 m and a distance of 20 m between the lowest point on the ground wire and the ground. The relevant information of these sites, including segment spacing and tower height, is shown in Table 4. Additionally, the geographical location marks of real sites A, B, and C are shown in Figure 8. In this paper, the length of the acquisition path is one segment, starting from the first power tower and ending at the second power tower in each site. The FPLIR walking speed is fixed at 0.1 m/s, resulting in different data acquisition times for each segment distance: 50 s, 86 s, 86 s, and 786 s.

#### 3.2.2. Range of Collection Angle Determination

A Zed 2i binocular camera was used for data collection and the view of the captured image was regulated by adjusting the rotation angle of the digital servos. It is important to note that small variations in the camera rotation angle can cause estimation failure, while excessive changes can result in an insufficient proportion of the power line being present in unbounded scene images. Therefore, when the FPLIR was moving along the ground wire, it was critical to determine the optimal capture angle range to avoid camera pose estimation failure or redundant data generation.

**Figure 9:**
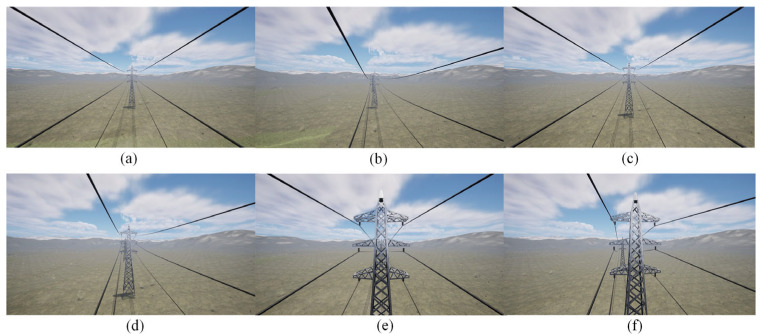
Field of view at partial angles in UE5: (**a**) angle I; (**b**) angle II; (**c**) angle III; (**d**) angle IV; (**e**) angle V; (**f**) angle VI.

**Figure 10:**
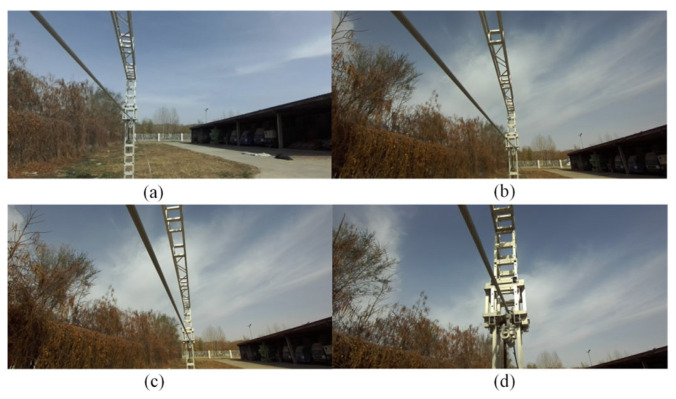
Field of view at partial angles from Site A: (**a**) angle I; (**b**) angle II; (**c**) angle III; (**d**) angle IV.

Different scenarios for the power transmission corridor were created in the Unreal Engine 5 (UE5) software, including plains, highways, grasslands, and mountains. The rotation ranges for the rocker arm camera were set individually. The range and sequence of the digital servo’s rotation angles were determined based on the distribution of PTLs within the camera’s field of view, as illustrated in Figure 9. Then, as the FPLIR rolled along the ground wire at Site A, the images captured by the camera were observed from different angles, as shown in Figure 10. Subsequently, image sequences from different ranges were established as datasets for pose estimation and comparison, using the technique described in Section 4.1. Finally, the optimal rotation ranges were determined to be between 70 and 110° in the rolling direction and 75 and 105° in the pitching direction. These ranges guarantee a high success rate in pose estimation for the generated progressive motion sequence images, while reducing duplication in sparse point clouds.

#### 3.2.3. Dataset Establishment

**Figure 11:**
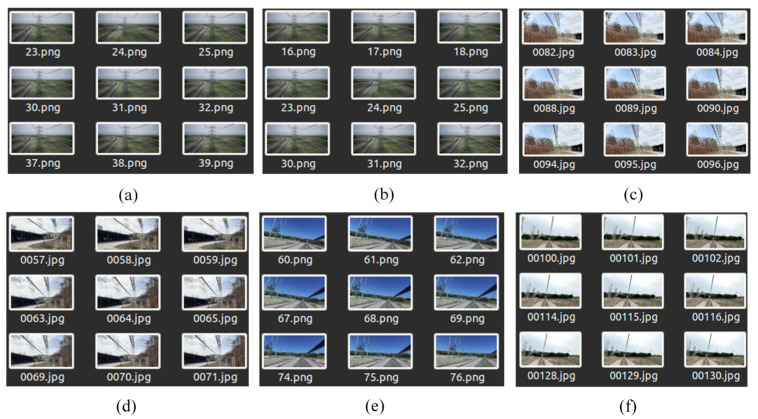
Some sequence images from datasets I to VI: (**a**) Set I; (**b**) Set II; (**c**) Set III; (**d**) Set IV; (**e**) Set V; (**f**) Set VI.

Two types of datasets were established: synthetic datasets (Datasets I and II) and real datasets (Datasets III to VI). Among them, Datasets III and IV were collected from Site A, Dataset V was from Site B, Dataset VI was from Site C, and Datasets I and II were from Site D. Figure 11 shows some images from the original Datasets I to VI. The four sets in the real dataset were all extracted from captured videos exported at a frequency of 2 frames per second, while the two sets in the synthetic dataset were extracted from videos exported at a frequency of 1 frame per second. Table 5 lists parameters such as the number of images and image clarity for each original dataset.

## 4. Methodology

**Figure 12:**
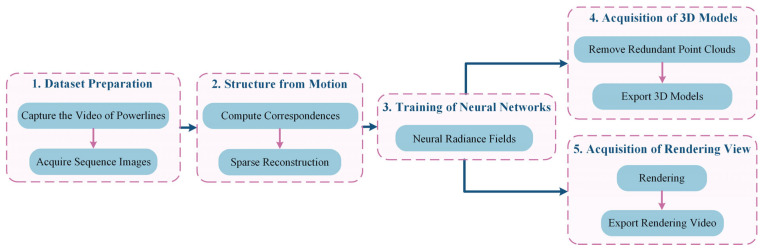
Working overflow.

The overall method consists of five stages, as depicted in Figure 12: (1) Dataset preparation stage, where videos of PTLs are recorded using a visual acquisition system and motion sequence images are exported to create basic datasets; (2) Structure from motion stage, involving the generation of a progressive motion sequence image dataset using algorithms, computation of image correspondence, extraction and matching of power line features, solving for camera poses, and obtaining a sparse point cloud model; (3) Neural network training stage, where new perspectives are synthesized and camera poses and sparse point clouds of PTLs are rendered using PL-NeRF; (4) 3D reconstruction model acquisition stage, which includes setting threshold space according to model requirements, removing redundant data, and exporting dense point cloud, meshes, and surfaces of PTLs; (5) Rendering effect acquisition stage, focused on rendering and exporting the video of PTLs from new perspectives.

The reconstruction of PTLs becomes increasingly challenging due to the unbounded nature of the scenes, the long and narrow characteristics of the power line, and the over-simplified texture feature. In this paper, a novel approach is proposed to address the above challenges. An improved NeRF method is combined with progressive motion sequence images to compensate for the gradual blurring or even disappearance of the target over long distances. The proposed method successfully reconstructs PTLs, with the particular details provided.

### 4.1. Estimation of Camera Poses

Accurate estimation of camera poses is crucial to improve the performance and accuracy of PTL reconstruction results. However, due to the unbounded nature of PTL scenes, accurately locating the on-board camera in the scene is challenging. Therefore, this paper adopts the Structure-from-Motion (SFM) technique [39] combined with our progressive motion sequence images to perform sparse reconstruction. This method enables the acquisition of sparse point clouds of PTLs and the estimation of camera poses.

**Figure 13:**
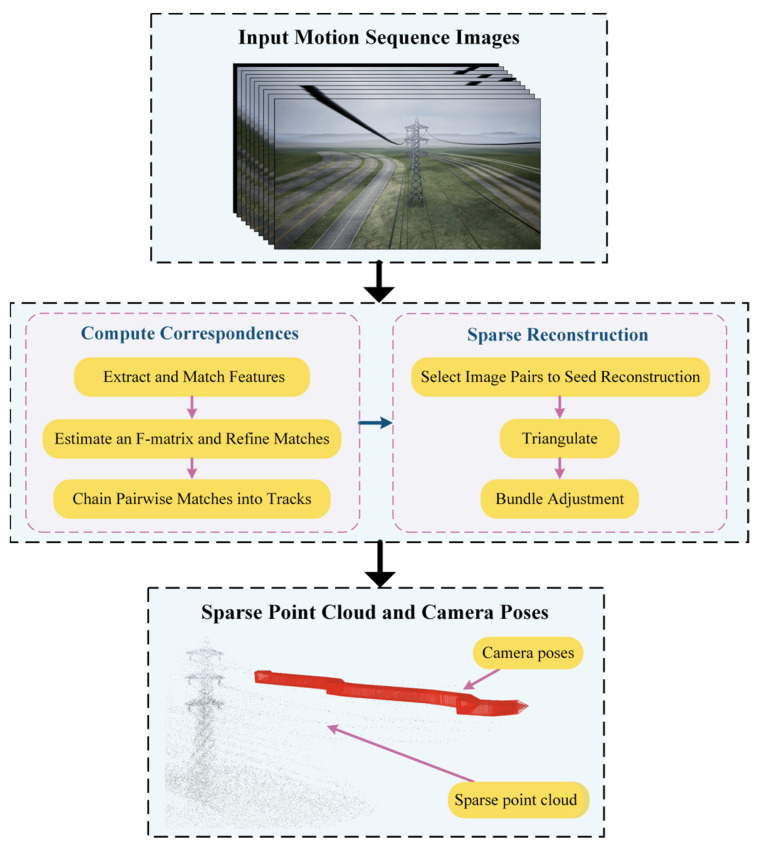
Principles of SFM technology.

Specifically, as illustrated in Figure 13, the principle of SFM begins with using the Scale-Invariant Feature Transform (SIFT) for feature extraction. Secondly, the random sample consistency (RANSAC) is employed to eliminate mismatched points during feature matching. Then, the basic matrix between adjacent sequence images is calculated by matching point pairs, and the matching pairs are optimized. Subsequently, the initial camera poses and motion trajectory are estimated. A selection process based on superior image correspondence pairs facilitates triangulation to generate a sparse 3D point cloud. Finally, relative pose estimation and global optimization techniques, such as Bundle Adjustment (BA), are used to estimate poses from all images.

The datasets presented in this paper are all motion sequence images, and the complete mathematical equation can be expressed as

(1) $$S=\left\{X^{\left(1\right)},X^{\left(2\right)},\cdots ,X^{\left(6\right)}\right\}$$

where any set of dataset samples $X^{\left(i\right)}$ is a two-dimensional sequence composed of two sequences $X_{1}^{\left(i\right)}$ and $X_{2}^{\left(i\right)}$ of length T, representing time and motion, respectively, which can be expressed as

(2) $$X_{1}^{\left(i\right)}=x_{11}^{\left(i\right)}x_{12}^{\left(i\right)}\cdots x_{1T}^{\left(i\right)},\;x\in R$$

(3) $$X_{2}^{\left(i\right)}=x_{21}^{\left(i\right)}x_{22}^{\left(i\right)}\cdots x_{2T}^{\left(i\right)},\;x\in E$$

After SFM, for each input dataset, we uniformly segment the motion and dynamically generate a new brightness field. Subsequently, we gradually introduce optimization for the subsequent frames. Specifically, whenever the estimated camera pose trajectory leaves the uncompressed space of the current brightness field, we initialize the new pose using the last frame of that trajectory:

(4) $${\left[R|t\right]}_{p+1}\leftarrow {\left[R|t\right]}_{p}$$

**Figure 14:**
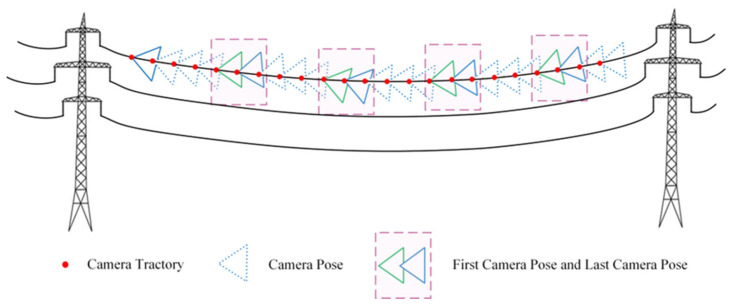
Progressive motion sequence.

The motion trajectory is segmented every five frames, with a camera pose added at the end of the segment. This introduces a local prior to ensure that the start pose of the next segment is close to the end pose of the previous segment. In Figure 14, triangles represent the camera pose at each moment in the motion sequence. Specifically, the last frame of the previous segment is represented by a green pose, while the first frame of the next segment is represented by the blue pose, thereby ensuring that the transition moments remain consistent. By extending the path and allowing smooth transitions, the sequence of progressive motion images increases the accuracy of pose estimation for power lines, resulting in a more complete reconstruction of PTLs.

**Figure 15:**
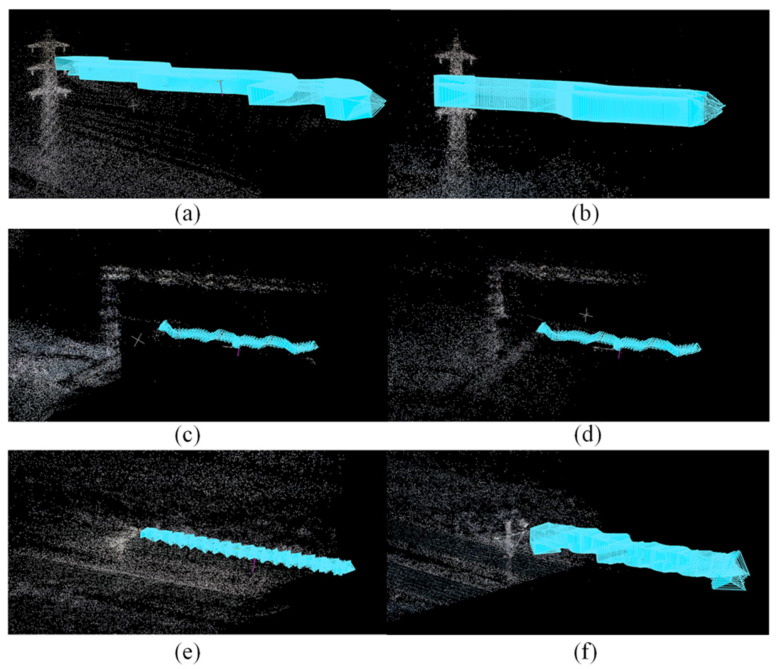
Sparse point clouds of PTLs and camera poses: (**a**) Set I; (**b**) Set II; (**c**) Set III; (**d**) Set IV; (**e**) Set V; (**f**) Set VI.

The above techniques are used to construct datasets consisting of progressive motion sequence images for estimating camera poses, thereby supporting the reconstruction of PTLs using PL-NeRF. Finally, it was confirmed that the use of an optimal rotation angle scheme for data collection can obtain good sparse point clouds of PTLs and improve the accuracy of camera poses. The findings illustrated in Figure 15 were instrumental in optimizing the data collection process, enhancing the data utilization, and improving the overall efficiency of reconstruction.

### 4.2. Neural Radiance Fields

#### 4.2.1. Preliminary Knowledge

**Figure 16:**
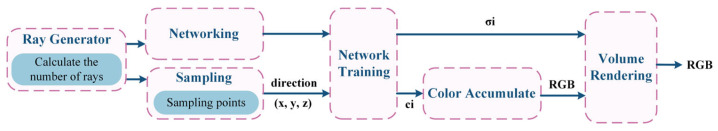
The pipeline of NeRF.

NeRF employs deep learning models to acquire the radiance field representation of scenes, which integrates volume rendering with implicit neural scene representation through multilayer perceptrons (MLPs). It comprises two main components: scene representation and rendering. In scene representation, a deep learning model is used to learn the radiance field of a 3D scene, including the intensity and color of the light emitted from each point in the scene. Specifically, the perceptrons represent the color and radiance intensity at specific locations within the scenery. For new scene points, their colors and radiance intensities are obtained through interpolation methods. Meanwhile, rendering involves using the acquired radiance field representation to generate a 3D reconstruction model. The overall workflow of NeRF, as shown in Figure 16, involves employing a ray-tracing algorithm to obtain the intersection point between the light emitted by the camera and the scene. It then uses the radiance field representation to calculate the color and light intensity at these points. Finally, by combining the color and intensity values from multiple sampling points, NeRF generates a 3D reconstruction model from the camera’s perspective.

**Figure 17:**
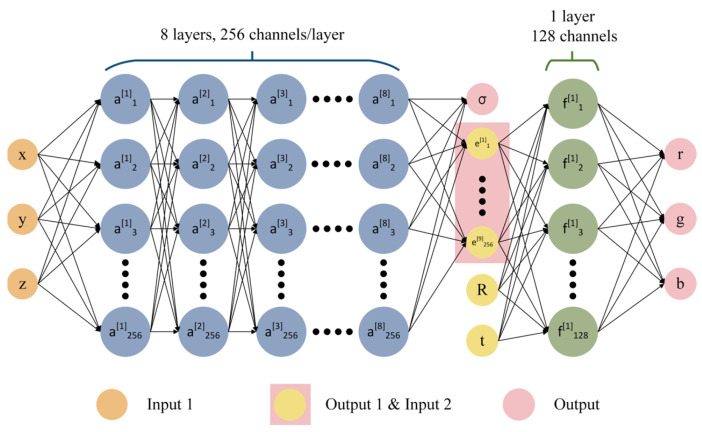
The principle of the multilayer perceptron in NeRF.

The principle of the MLP for NeRF 3D reconstruction is shown in Figure 17. A fully connected network was used to approximate represent this continuous 5D scene:

(5) $$F_{\Theta }:\;\left(x,d\right)\to \left(c,\sigma \right)$$

Firstly, the overall input of $F_{\Theta }$ is the 5D coordinates $\left(x,y,z,\theta_{i},\phi \right)$, where x=(x,y,z) is the 3D coordinate in the 3D scene and $d=\left(\theta_{i},\phi \right)$ is the camera perspective direction. The final output is the 4D vector (R,G,B,σ), which is the color value c=(R,G,B) and opacity σ. The whole principle can be divided into two parts. In the first part, the input consists of (x,y,z), which is passed through eight fully connected layers with a ReLU activation function, where each layer has 256 channels for learning, and the output consists of the opacity σ and a 256-dimensional feature vector. In the second part, the input consists of the 256-dimensional feature vector obtained from the output of the first part, together with the camera view direction $d=\left(\theta_{i},\phi \right)$. These inputs are passed through a fully connected layer with a ReLU activation function and 128 channels for learning. The final output is an RGB color value c. The opacity σ represents the probability of the ray ending at the point x after reaching it. The camera ray at the point can be represented as

(6) r(t)=o+td

Among these, o is the 3D coordinate point and t is the actual physical length. The expected color at the point can be obtained as follows:

(7) $$C\left(r\right)=\int_{t_{n}}^{t_{f}}T\left(t\right)\sigma \left(r\left(t\right)\right)c\left(r\left(t\right),d\right)dt$$

where $t_{n}$ represents the nearest boundary and $t_{f}$ represents the farthest boundary. T(t) represents the cumulative transmittance of light rays along rays $t_{n}$ to t, which can be obtained via the following equation:

(8) $$T\left(t\right)=\exp\left(-\int_{t_{n}}^{t}\sigma \left(r\left(s\right)\right)ds\right)$$

However, the MLP is limited to querying fixed discrete positions, which often restricts the resolution when rendering discrete voxel grids using deterministic sampling. Therefore, a stratified sampling approach is used to divide the $\left(t_{n},t_{f}\right)$ into N equal-sized bins, and then randomly selects one sample from each bin uniformly, allowing the MLP to evaluate continuous positions during the optimization and render continuous scenes. These samples are used to estimate C(r):

(9) $$\hat{C}\left(r\right)=\overset{N}{\underset{i=1}{\sum }}T_{i}\left(1-\exp\left(-\sigma_{i}\delta_{i}\right)\right)\;c_{i}$$

where $\delta_{i}=t_{i+1}-t_{i}$ represents the physical distance between adjacent samples.

(10) $$T_{i}=\exp\left(-\overset{i-1}{\underset{j=1}{\sum }}\sigma_{j}\delta_{j}\right)$$

The above equations are the core content of NeRF, but there are some issues with rendering models, such as blurring, lack of detail, and too many invalid sample points. Therefore, the original NeRF method added the PE and the hierarchical sampling strategy to improve the problems:

(1) Position Encoding

Deep learning models exhibit strong nonlinear expressions when dealing with spatial information, but, without proper encoding methods, spatial information can be easily lost. Specifically, sine and cosine functions have periodicity, and, in deep learning, it is often easier to learn low-frequency functions than high-frequency ones. As a result, deep networks tend to focus on learning low-frequency functions in space while losing high-frequency information. To address this issue, NeRF employs PE as a specialized mapping technique, in which low-frequency information is transformed into high-frequency information using high-frequency functions that are then fitted by the deep network. In other words, coordinate representations are initially transformed into a higher-dimensional space before being used as input for the MLP, increasing the dimensionality of the data and, thus, improving the accuracy and performance of the model.

$F_{\Theta }=F_{\Theta }^{\prime }\circ \gamma$ is redefined in this paper, where γ represents the mapping from ℝ to a high-dimensional space ${\mathbb{R}}^{2L}$ and $F_{\Theta }^{\prime }$ is a regular MLP. The encoding function employed is achieved by multi-period sine and cosine functions:

(11) $$\gamma \left(p\right)=\left(\sin\left(2^{0}\pi p\right),\cos\left(2^{0}\pi p\right),\cdots ,\sin\left(2^{L-1}\pi p\right),\cos\left(2^{L-1}\pi p\right)\right)$$

where γ(·) is applied separately to the three coordinate values in x and the camera viewing direction d. In NeRF, L=10 is set for x and L=4 is set for d, which allows MLP to approximate high-frequency functions more effectively;

(2) Hierarchical Sampling Strategy

The rendering strategy of NeRF involves densely evaluating the neural radiance field network at N query points along each camera ray. Due to the different contributions of diverse regions to the final color output, uniformly sampling each ray can lead to numerous invalid points, including duplicate sampling of free space and occluded areas that do not contribute to the rendered image, thereby reducing sampling efficiency. Therefore, a layered sampling strategy is proposed, which involves intensive sampling in areas with significant contributions and limited or no sampling in areas with minimal contributions. The strategy involves the simultaneous optimization of two networks, namely, a ‘coarse’ network and a ‘fine’ network, rather than relying on just one network to represent the scene. In the case of the ‘coarse’ network, we uniformly sample $N_{c}$ points along each ray and calculate the color weighting values corresponding to each sampling point according to the following equation:

(12) $${\hat{C}}_{c}\left(r\right)=\overset{N_{c}}{\underset{i=1}{\sum }}\varpi_{i}C_{i}$$

where,

(13) $$\varpi_{i}=T_{i}\left(1-\exp\left(-\sigma_{i}\delta_{i}\right)\right)$$

To generate a PDF that produces segmented constants along the ray and normalize them,

(14) $${\hat{\varpi }}_{i}=\frac{\varpi_{i}}{\sum_{j=1}^{N_{c}}\varpi_{j}}$$

Subsequently, the inverse transform sampling technique is employed to select high probability density points from this distribution function as the second set of sampling points for reweighted sampling. Finally, the first set of $N_{c}$ samples is combined with the second set of $N_{f}$ samples to evaluate the “fine” network, which calculates the final rendered color of the ray using Equation (8).

Due to the requirement of a large amount of rendered image data in supervised learning, NeRF faces a high demand for image data. When dealing with large-scale scenes such as PTLs, the discontinuity in the PTL model results in jagged edges and slow rendering speed. Therefore, this study proposes PL-NeRF combined with progressive motion sequence images of PTLs to achieve faster reconstruction of high-quality PTLs.

#### 4.2.2. PL-NeRF

This study integrates several published neural radiance field methods, mainly influenced by Mip-Nerf [34] and Instant-ngp [37], along with other reference methods such as NeRF- [40], NeRF W [50], and Ref NeRF [51]. Our objective is to simplify the training, optimization, and rendering processes of NeRF. To effectively reconstruct PTLs, we have developed a method called PL-NeRF, which is combined with our progressive motion sequence images.

Due to the thin and low-texture features of power lines, using NeRF results in discontinuity, jagged edges, and slow rendering speed during the power line reconstruction. To address these issues and reconstruct a continuous PTL model, this paper proposes two improvements based on the original NeRF: (1) PE method; (2) compression method for scene reconstruction space.

The original NeRF method employs the PE technique, which projects an infinitesimally small amount of light onto each pixel and constructs position-encoded features from a given point in space. However, this approach results in a large sample size and significant data aliasing issues, leading to ghosting and discontinuity problems in the power line reconstruction model. To address these challenges, this study combines IPE and HE techniques to separately encode directional and positional information. This approach effectively reduces the number of samples along the beam, generating anti-aliasing features.

Regarding spatial compression, the original NeRF uses NDC, which defines only the nearest and farthest sample points along the optical axis. In the case of unbounded real PTL scenes, these boundaries are not well-defined, complicating the process of determining a stopping point for sample processing. To address this in unbounded scenes, there are typically two solutions: increasing the distance for far sampling or transforming the space into a fixed volume. In this study, considering the specific structural conditions of PTLs, the space compression method is adopted to twist the space into a fixed volume, mainly inspired by Mip-Nerf 360 [44]. However, unlike Mip-Nerf 360, which uses the norm to compress into a sphere, we apply the norm to compress into a cube shape. This adaptation allows for better alignment with hash encoding and is more suitable for structured working conditions.

**Figure 18:**
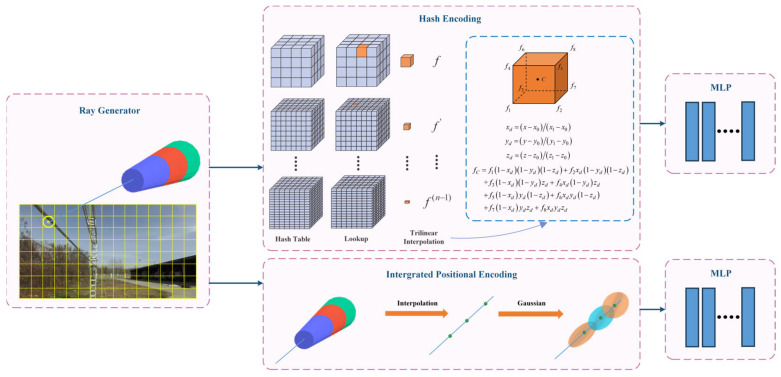
PL-NeRF field.

**Figure 19:**
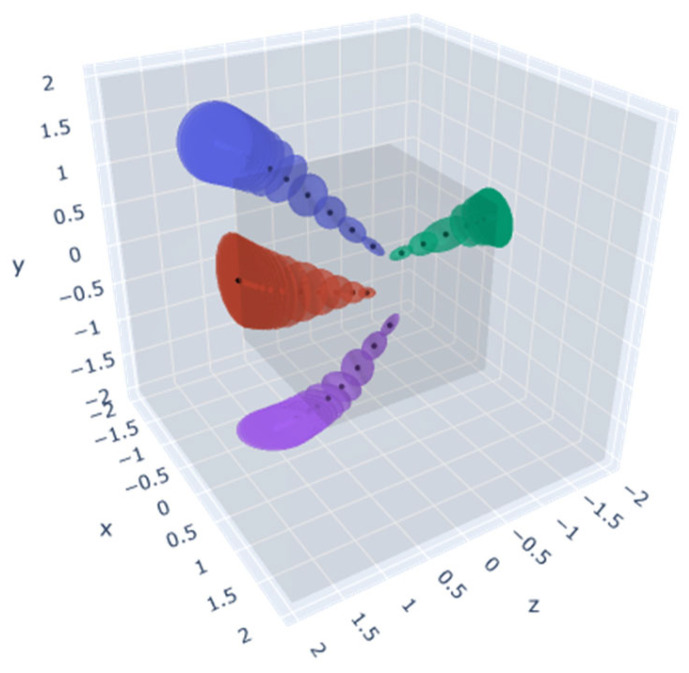
Contraction: Using an $L^{\infty }$ norm.

The PL-NeRF field is shown in Figure 18. The first step is to generate ray bundles based on the number of pixels in the input image. For each pixel, a cone beam is emitted by the camera and then divided into frustums perpendicular to its axis. In the second step, HE and IPE are used to encode position and direction information, respectively. Finally, in the third step, the encoded information is fed into the respective MLP network. During the HE, the scene space is first normalized using the norm illustrated in Figure 19, where each small vertex has quantified coordinates, and the Hash Table is initialized. Subsequently, a Hash Function is constructed to establish an index for each small vertex coordinate in the Hash Table. For a given input, we determine its associated small vertices and employ the Hash Function to locate their corresponding indices in the Hash Table, from which the values are retrieved and utilized for cubic interpolation calculations. Once these interpolations are obtained, they are linked together and passed into an MLP network. The IPE stage involves finding the truncated frustum region and integrating the PE of the viewing cone region. Then, we approximate the integration using a multivariate Gaussian approach and compute the Gaussian representation of the truncated frustum to obtain a multivariate Gaussian representation of encoding. Finally, we truncate the frustum’s encoding to calculate the expected encoding, which is transmitted to another MLP network. This process results in a representation that more accurately reflects the average position and depth variations within the area. Consequently, it improves our model’s global understanding and representation of local scene details.

**Figure 20:**
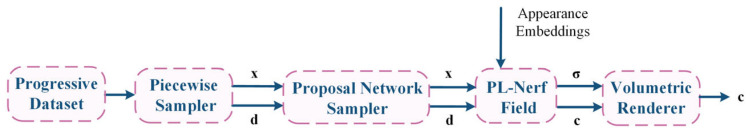
The pipeline of PL-NeRF.

After implementing the above improvements, this method effectively reconstructs continuous PTLs, thus improving the reconstruction quality and efficiency of PTLs. In addition, Figure 20 demonstrates the introduction of the proposal network sampler, appearance embedding technology, and volume renderer in this paper. Among them, the proposal network sampler merges the sampling positions into the area in the scene where the first surface intersects, which contributes the most to the final rendering and further improves the reconstruction quality of the PTLs. The appearance embedding technology employs an image-by-image approach to embed the appearance information into the neural radiance field, taking into account the exposure differences of the training camera, which enables a better representation of texture and color details in the PTLs. Finally, the volume renderer for volume rendering also incorporates techniques from Ref-NeRF [51] to calculate and predict the normal, convert neural radiance fields into visualized 3D scenes and generate high quality RGB rendered images.

In summary, PL-NeRF utilizes techniques such as truncated cone sampling, unbounded space contract compression, hash encoding, proposal network sampler, appearance embedding, and volume rendering combined with the progressive motion sequence images of PTLs in this study to achieve 3D reconstruction of PTLs.

## 5. Experiments

The experiments detailing the algorithm in this chapter employ a dataset of PTLs, as previously established in Section 3.2.3, using three highly correlated 3D reconstruction methods (F^2^-NeRF, Instant-ngp, and Volinga), along with the proposed PL-NeRF method. Furthermore, a comparative analysis of these methods was conducted, based on their reconstruction results, to confirm the feasibility and effectiveness of the proposed approach.

### 5.1. Experimental Settings

Based on the sparse point cloud and camera poses obtained in Section 4.1, the rendering training of the PTLs is started. The main hardware and software parameters of the testing computer used in this study are outlined below:

(1) Computing host: CPU—Intel (R) Core (TM) i9 12,900 K, RAM64 G, GPU—NVIDIA GeForce RTX 3090 24 G;

(2) Environment: Ubuntu 18.04, PyTorch 1.12.1, CUDA 11.3, Python 3.8.

The construction of the original datasets is described in Section 3.2.3. The pose calculation steps involve screening out images with blur, failed feature matching, and inability to calculate poses. Subsequently, the remaining images are selected as the final experimental dataset according to the following distribution: 80% for training, 10% for validation, and 10% for testing purposes. Table 6 presents the distribution of images in each experimental dataset.

### 5.2. Evaluation Metrics

This paper uses four evaluation metrics, namely, Peak Signal to Noise Ratio (PSNR), Structural Similarity Index (SSIM), Learned Perceptual Image Patch Similarity (LPIPS), and Frames Per Second (FPS). PSNR, SSIM, and LPIPS are employed to evaluate the fidelity of the reconstruction model, while FPS is used to evaluate the rendering speed in 3D reconstruction.

#### 5.2.1. PSNR

PSNR refers to the average difference between the maximum signal and background noise in an image at the peak signal level, which is measured in decibels (dB). A higher value of PSNR indicates a greater similarity between the reconstructed model and the original image, thus reflecting a higher image quality. The PSNR can be computed as follows:

(15) $$PSNR=10\log_{10}\left(\frac{{\left(2^{n}-1\right)}^{2}}{MSE}()\right)$$

where n is the number of bits per sampled value and MSE is the mean square error of the original image after 3D reconstruction.

#### 5.2.2. SSIM

SSIM is an indicator used to measure the similarity between the reconstructed model and the original image. Compared with PSNR, it is more suitable for evaluating image quality in accordance with human visual characteristics. Given two images x and y, the calculation formula for SSIM is

(16) $$SSIM(x,y)=\frac{\left(2\mu_{x}\mu_{y}+c_{1}\right)\left(2\sigma_{xy}+c_{2}\right)}{\left(\mu_{x}{}^{2}+\mu_{y}{}^{2}+c_{1}\right)\left(\sigma_{x}{}^{2}+\sigma_{y}{}^{2}+c_{2}\right)}$$

where $\mu_{x}$ and $\mu_{y}$ are the average values of x and y, respectively, seen as estimates of the brightness; $\sigma_{x}{}^{2}$ and $\sigma_{y}{}^{2}$ are the variance of x and y, respectively, used as an estimate of contrast; and $\sigma_{xy}$ is the covariance of x and y as a measure of structural similarity. $c_{1}$, $c_{2}$, and $c_{3}$ are constants used to avoid denominators of 0, usually obtained from the following equations:

(17) $$c_{1}={\left(k_{1}L\right)}^{2}$$

(18) $$c_{2}={\left(k_{2}L\right)}^{2}$$

(19) $$c_{3}=\frac{1}{2}c_{2}$$

Among them, L is the dynamic range of pixel values, and other values are usually taken as $k_{1}=0.01$, $k_{2}=0.03$. The range of SSIM values is −1 to 1, and, in practical applications, the similarity value is typically normalized to a range of 0 to 1, with higher values indicating greater structural similarity between the two images. When the SSIM value is equal to 1, the two images are identical.

#### 5.2.3. LPIPS

The metric LPIPS, referred to as perceptual loss, is a deep learning-based image quality evaluation metric that is more closely aligned with human perception than traditional methods such as L2/PSNR, SSIM, and FSIM. It estimates the difference between two images by calculating the distance metric between features. Given the original image block x and the modeled image block $x_{0}$, the calculation formula for LPIPS is as follows:

(20) $$d\left(x,x_{0}\right)=\underset{l}{\sum }\frac{1}{H_{l}W_{l}}\underset{h,w}{\sum }{\left||\omega_{l}⊙\left({\hat{y}}_{hw}^{l}-{\hat{y}}_{0hw}^{l}\right)|\right|}_{2}^{2}$$

where d is the distance between x and $x_{0}$. The model extracts features from layer l and normalizes units in the channel dimension, recording the result as ${\hat{y}}^{l},\;{\hat{y}}_{0}^{l}\in \;{\mathbb{R}}^{H_{l}\times W_{l}\times C_{l}}$. It scales the active channel using vector $w_{l}\in {\mathbb{R}}^{C_{l}}$ and calculates the distance $l_{2}$, then takes the average value in space and sums it on the channel. The similarity between the two images increases as the value of LPIPS decreases, and, conversely, the dissimilarity becomes greater as LPIPS increases.

#### 5.2.4. FPS

FPS is the number of frames that the system can process per second; it is used to measure the smoothness and real-time performance of model rendering and can also be used to evaluate system performance. It is calculated from the ratio of rendering time and rendered frames:

(21) $$FPS=\frac{1}{T_{s}}$$

where $T_{s}$ is to the time required to render one frame of an image, usually in milliseconds (ms). The reciprocal of the rendering time divided by the number of frames gives the number of frames per second. A higher value of FPS indicates that the system can process images or render scenes more quickly, making the scene feel more realistic and providing a smoother interactive experience. By monitoring changes in FPS, the impact of parameter settings on performance can be determined, and adjustments and optimizations can be made to achieve a better balance.

### 5.3. Experimental Results

**Figure 21:**
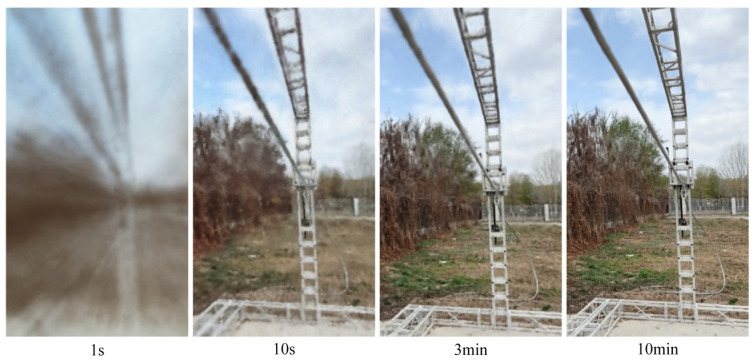
Rendering process.

**Figure 22:**
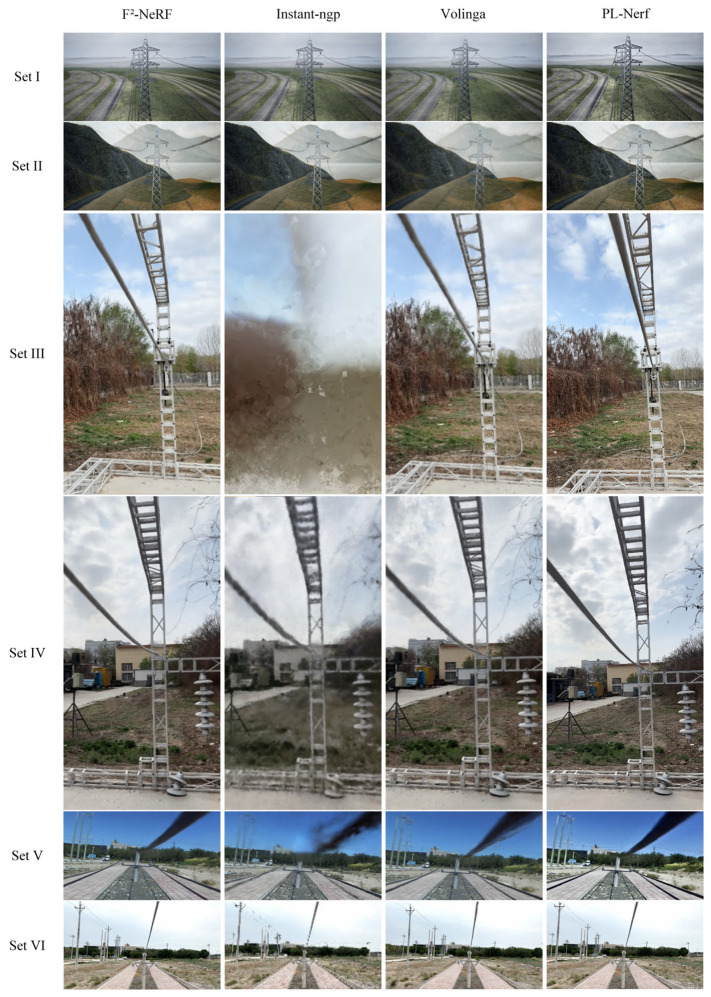
Comparison of the effects of four algorithms.

**Figure 23:**
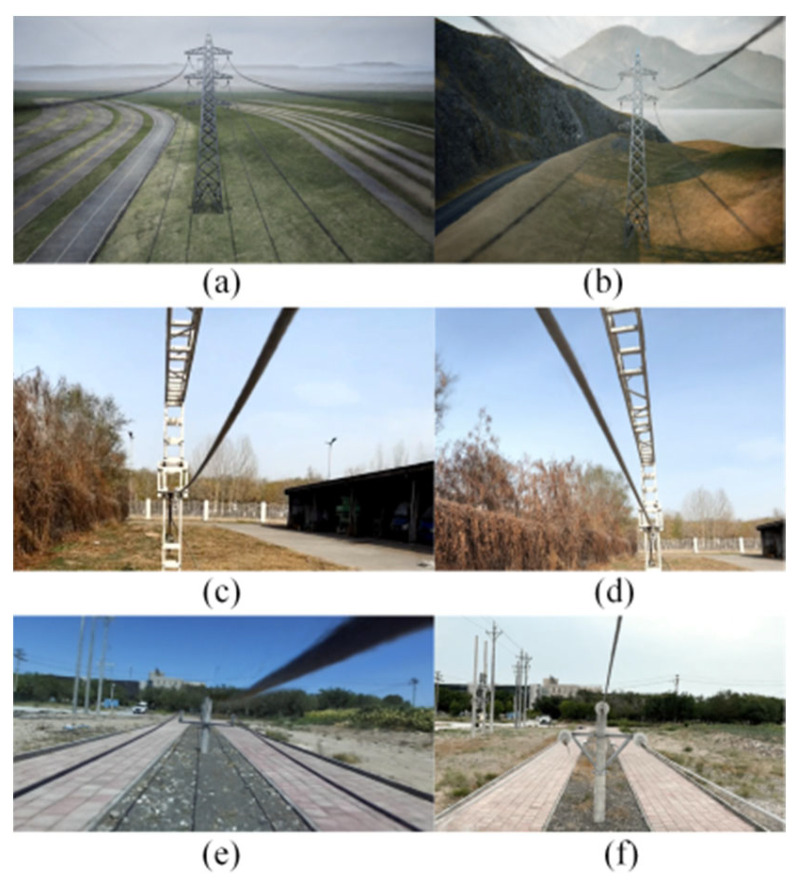
Rendered results: (**a**) Set I; (**b**) Set II; (**c**) Set III; (**d**) Set IV; (**e**) Set V; (**f**) Set VI.

The dataset is trained through a process in which the model first enters a fast convergence stage, then gradually converges until it reaches a stable state. The image gradually becomes clearer, and the rendering effect of power line details gradually improves. The real-time rendering effect of dataset III during the training process is shown in Figure 21. The rendering performance of each algorithm in datasets I to VI is shown in Figure 22, and the final rendering result is shown in Figure 23. The last row of Table 7 lists the four evaluation indicators obtained from all training results using this algorithm.

## 6. Discussion

### 6.1. Effect of Select Methods

In Section 5.1, a comparison is made between three relevant reconstruction methods and the proposed methods based on our datasets. These include F^2^-NeRF, which is a neural radiance field method for reconstructing scenes from arbitrary motion trajectories, Instant-ngp, which is an interactive real-time reconstruction technique that allows for dynamic modifications, and Volinga, which is a fast reconstruction technique specifically designed for virtual datasets. The results of the neural radiance field method used for 3D reconstruction from progressive motion sequence images of PTLs are shown in Table 7. The relationship between the values of the four evaluation metrics and their performance has been detailed in Section 5.2, and the best performances are highlighted in bold and underlined. Among the obtained reconstruction metrics, the PL-NeRF method outperforms other methods, with the exception of FPS. The PL-NeRF method prioritizes the improved performance of reconstructed models over rendering smoothness and real-time capabilities. As for Volinga, it focuses more on speed than quality. Figure 22 demonstrates that the visual effect achieved with PL-NeRF is the most realistic. Overall, the PL-NeRF method provides the best reconstruction effect for 3D reconstruction of PTLs compared to the other algorithms mentioned.

Furthermore, compared to traditional 3D reconstruction techniques, radiance field-based neural methods show significant advancements in both scene accuracy and speed of 3D reconstruction. This study proposes PL-NeRF, an improved NeRF method, for the reconstruction of PTLs in unbounded scenes. Combined with the established progressive motion sequence images, some favorable results are finally obtained.

Table 8 compares traditional methods and deep learning approaches, focusing on their visual effects, modelling efficiency, and technical value. The proposed method outperforms, both in efficiency and model details, for PTL reconstruction, while reducing the associated cost in PTL scenes. This paper offers a practical approach to 3D reconstruction of PTLs, providing a useful guide for digital automatic inspection during the planning, design, construction, and maintenance of transmission corridors.

### 6.2. Motivation and Contributions

Previous studies on 3D reconstruction of power lines have predominantly relied on LiDAR techniques, which incur significant equipment costs and are often hindered by obstructions, limiting their widespread adoption. Our study proposes to overcome these challenges by adopting a progressive motion sequence images approach, which reduces equipment costs and lessens the impact of environmental factors.

Given that the background of PTLs often fails within an unbounded scene, capturing intricate details of distant objects becomes challenging, often resulting in blurred reconstructions. To address this, we have introduced a position-encoding method within the PL-NeRF framework. This method effectively translates low-frequency information into high-frequency details using high-frequency functions. Incorporating this approach into a deep network framework allows for a more comprehensive representation of PTLs’ intricate details. We have also implemented segmented and network samplers, which efficiently sample both distant and nearby objects. These samplers merge their positions within the most influential areas of the scene for final rendering, thereby enhancing the quality of the reconstruction.

Moreover, considering the inherent characteristics of the power line, which is thin and lacks texture, feature point matching becomes a challenge. To solve this, this study uses a developed visual acquisition system to collect multi-view images of PTLs at close distances, creating progressive motion sequence datasets. This approach effectively addresses the issue of incomplete reconstruction models due to failed pose estimation over long distances.

### 6.3. Limitations and Future Work

The proposed method has certain limitations, including the possibility of improving evaluation metrics, non-real-time completion of 3D reconstruction of PTLs during inspection, and the need to improve the accuracy of explicit reconstruction models. Additionally, PL-NeRF is based on a relatively complete continuous dataset, that is, it assumes that there are no obstacles on the ground wire that require the FPLIR to fly over them. These areas require further investigation and continuous refinement in future studies.

Firstly, the improvement of evaluation metrics mainly involves the consideration of hyperparameter tuning (e.g., learning rate, optimizer selection), preprocessing techniques (e.g., denoising, background removal), and the refinement of neural network structure designs. Secondly, with regard to real-time 3D reconstruction of PTLs, the performance requirements for hardware devices are relatively high, along with the consideration of sensor configurations and algorithms suitable for achieving real-time 3D reconstruction. Finally, to further improve the accuracy of the explicit model of PTLs, it is necessary to consider how to build a new network model to construct the explicit model after the implicit model training is completed. It is possible to improve the results by merging multi-sensor information fusion with LiDAR equipment or by fitting the catenary structure of the power line.

## 7. Conclusions

To address the image-based 3D reconstruction difficulties in PTLs, this paper proposes PL-NeRF, an improved NeRF method for 3D reconstruction that combines the novel progressive motion sequence images of PTLs. The main results of this paper are summarized as follows:

(1) The motion sequence images acquired in the optimal range of rotation angles were equally divided in each motion segment. This approach dynamically generates a novel neural radiance field, which ensures that the initial pose of each subsequent segment converges towards the final pose of the preceding segment. Consequently, this establishes progressive motion sequence images for PTLs, enabling the successful estimation of long-term trajectory poses using this dataset;

(2) The spatial compression and encoding methods for scene reconstruction were refined from the original NeRF approach to better suit the narrow and elongated spatial structure of overhead transmission corridors as well as the continuity requirements of power lines. Specifically, an $L^{\infty }$ norm scene contraction method, in conjunction with IPE and HE methods in encoding, was employed to improve both the quality and efficiency of the power line reconstruction;

(3) A comparative experiment was conducted to assess the reconstruction effect of the proposed method with three highly correlated radiance field-based neural methods. With the exception of the FPS metric, the PL-NeRF method exhibited commendable performance in the final 3D reconstruction results, with fidelity metrics reaching PSNR = 29, SSIM = 0.871, and LPIPS = 0.087. These results, except for the FPS metric, confirm the practicality and effectiveness of the proposed PL-NeRF method.

In our future research, we will continue to optimize the neural network architecture to improve both the speed and fidelity of model reconstruction. In addition, we intend to explore the use of a catenary structure to fit the power line model, with the aim of achieving a more accurate PTL representation.

## Figures and Tables

**Table 2:** Collection methods for multi-view images.

Camera Trajectory	Pros and Cons
Free	Slow rendering and poor reconstruction of complex scene details, but require complex algorithms and processes.
Forward-facing	Simple trajectory, small amount of data, leading to missing some information.
Object-centric	Can provide complete geometric information about an object, but take longer, cost more, and require more storage and processing resources.

**Table 3:** Collection platform-related parameters.

Device	Digital Servo 1	Digital Servo 2	Camera
Field range/°	75~105	70~110	110 × 70 × 120 (H × V × D)
Depth/m	/	/	0.3~20
Resolution	/	/	(2208 × 1242) @15fps

**Table 4:** The relevant information of the four sites.

	Information	Length/(m)	Height of the Power Tower/(m)	Collecting Time/(s)
Sites				
Site A		6.4	3	50
Site B		10	1.5	86
Site C		10	2.0	86
Site D		80	30	786

**Table 5:** The related parameters of images.

	Parameters	Length/(m)	Height of the Power Tower/(m)
Datasets			
Set I		1572	2048 × 1080
Set II		1572	2048 × 1080
Set III		200	1920 × 1080
Set IV		200	1920 × 1080
Set V		344	1920 × 1080
Set VI		344	1920 × 1080

**Table 6:** The distribution of rest images.

	Parameters	Rest	Training	Validation	Test
Datasets					
Set I		720	576	72	72
Set II		606	484	61	61
Set III		186	148	19	19
Set IV		168	134	17	17
Set V		340	272	34	34
Set VI		332	266	33	33

**Table 7:** Comparison between four algorithms.

		Set I	Set II	Set III	Set IV	Set V	Set VI
**F^2^-NeRF**	**PSNR**	21.76	20.88	18.57	21.65	24.24	22.13
	**SSIM**	0.625	0.709	0.626	0.618	0.747	0.734
	**LPIPS**	0.447	0.515	0.451	0.319	0.226	0.119
	**FPS**	1.153	1.824	1.173	0.843	0.918	**1.855**
**Instant-ng** **p**	**PSNR**	21.33	19.06	16.34	15.26	23.18	20.39
	**SSIM**	0.552	0.539	0.396	0.435	0.783	0.773
	**LPIPS**	0.628	0.636	0.707	0.809	0.279	0.191
	**FPS**	0.205	0.203	0.196	0.196	0.374	1.547
**Volinga**	**PSNR**	24.25	22.94	20.46	21.96	22.20	19.17
	**SSIM**	0.724	0.69	0.625	0.700	0.786	0.533
	**LPIPS**	0.322	0.428	0.290	0.256	0.253	0.179
	**FPS**	**2.342**	**2.275**	**2.427**	**2.432**	**2.489**	1.755
**PL-NeRF**	**PSNR**	**28.59**	**29.00**	**26.340**	**26.411**	**26.26**	**27.58**
	**SSIM**	**0.837**	**0.871**	**0.846**	**0.864**	**0.814**	**0.883**
	**LPIPS**	**0.057**	**0.087**	**0.070**	**0.077**	**0.086**	**0.047**
	**FPS**	0.408	0.403	0.434	0.433	0.435	0.443

The relationship between the values of the four evaluation metrics and their performance has been detailed in Section 5.2, and the best performances are highlighted in bold and underlined.

**Table 8:** Comparison between traditional methods and deep learning methods.

	Traditional Methods		Deep Learning Methods
	SFM	Lidar	Ours
Visual effect	Average	Good	Best
Speed	Slow	Slow	Fast
Morphology	Valid	Valid	Excellent
Textures	Invalid	Invalid	Valid
Noise	Few	Many	Less
Lighting requirement	High	Low	Low
Trajectory deviation	Robust	Sensitive	Robust
Price	Cheap	Expensive	Cheap

## Data Availability

Data are contained within the article.
